# The identification of cognitive impairment in Parkinson’s disease using biofluids, neuroimaging, and artificial intelligence

**DOI:** 10.3389/fnins.2024.1446878

**Published:** 2024-09-26

**Authors:** Anthaea-Grace Patricia Dennis, Antonio P. Strafella

**Affiliations:** ^1^Krembil Brain Institute, University Health Network (UHN), Toronto, ON, Canada; ^2^Institute of Medical Science, Temerty Faculty of Medicine, University of Toronto, Toronto, ON, Canada; ^3^Brain Health Imaging Centre, Centre for Addiction and Mental Health, Campbell Family Mental Health Research Institute, Toronto, ON, Canada; ^4^Morton and Gloria Shulman Movement Disorder Unit, Edmond J. Safra Parkinson Disease Program, Division of Neurology, Department of Medicine, Toronto Western Hospital, University Health Network, Toronto, ON, Canada

**Keywords:** cognitive impairment, Parkinson’s disease, diagnosis, biomarkers, machine learning, artificial intelligence

## Abstract

**Introduction:**

Parkinson’s disease (PD) is a neurodegenerative movement disorder causing severe disability and cognitive impairment as the disease progresses. It is necessary to develop biomarkers for cognitive decline in PD for earlier detection and prediction of disease progression.

**Methods:**

We reviewed literature which used artificial intelligence-based techniques, which can be more sensitive than other analyses, to determine potential biomarkers for cognitive impairment in PD.

**Results:**

We found that combining biomarker types, including those from neuroimaging and biofluids, resulted in higher accuracy. Focused analysis on each biomarker type revealed that using structural and functional magnetic resonance imaging (MRI) resulted in accuracy and area under the curve (AUC) values above 80%/0.80, and that beta-amyloid-42 and tau were able to classify PD subjects by cognitive function with accuracy and AUC values above 90%/0.90.

**Discussion:**

We can conclude that applying both blood-based and imaging-based biomarkers may improve diagnostic accuracy and prediction of cognitive impairment in PD.

## 1 Overview of Parkinson’s disease and mild cognitive impairment in PD

Parkinson’s disease (PD) is one of the most common movement disorders and neurodegenerative disorders, affecting millions of adults over 65 years of age (Marras et al., 2018; Song et al., 2022). The most common feature of PD is Parkinsonism; symptoms of Parkinsonism include bradykinesia, resting tremor, and muscular rigidity. As the disease progresses, the disease can affect cognitive function, causing cognitive decline (Mihaescu et al., 2022). This cognitive decline can range from mild cognitive impairment (MCI) to dementia (within PD, this is referred to as PD dementia or PDD). Litvan et al. (2011) found that 26.7% of PD patients without dementia have MCI and that the incidence of MCI increases with disease duration, disease severity, and age. Other studies found that 21–24% of PD patients have MCI at time of diagnosis (Lawson et al., 2017) and over 80% of patients with PD will develop PDD (Hely et al., 2008).

To diagnose a PD patient as having MCI, a gradual decline in cognitive ability must be noted by the patient, informant, or clinician. Additionally, cognitive deficits must not significantly interfere with the patient’s functional independence must be detectable through either a “formal neuropsychological testing or a scale of global cognitive abilities” (Litvan et al., 2012). One commonly applied assessment of global cognitive abilities in both research and clinical practice is the Montreal Cognitive Assessment (MoCA). The MoCA is a one-page test out of 30 points, which covers executive function, memory, visuospatial ability, language, and attention (Bakeberg et al., 2020). The most common presentations of MCI in PD are executive function, visuospatial ability, and language (Bakeberg et al., 2020). As there are many different presentations of cognitive impairment and multiple cognitive assessments may be required to evaluate global cognitive performance, MCI can be difficult to detect (Bakeberg et al., 2020). It is unclear why some PD patients develop MCI or PDD and others do not, and specifically what influences different presentations of MCI (Bakeberg et al., 2020). Additionally, the risk of progression from PD-MCI to PDD increases as patients age (Bakeberg et al., 2020); early detection of MCI is therefore essential. To improve diagnosis of MCI in PD, it is necessary to determine what prospective indicators or biomarkers have succeeded in prior research for onset of MCI in PD (FDA-NIH Biomarker Working Group, 2016).

## 2 Methodology

**Figure 1:**
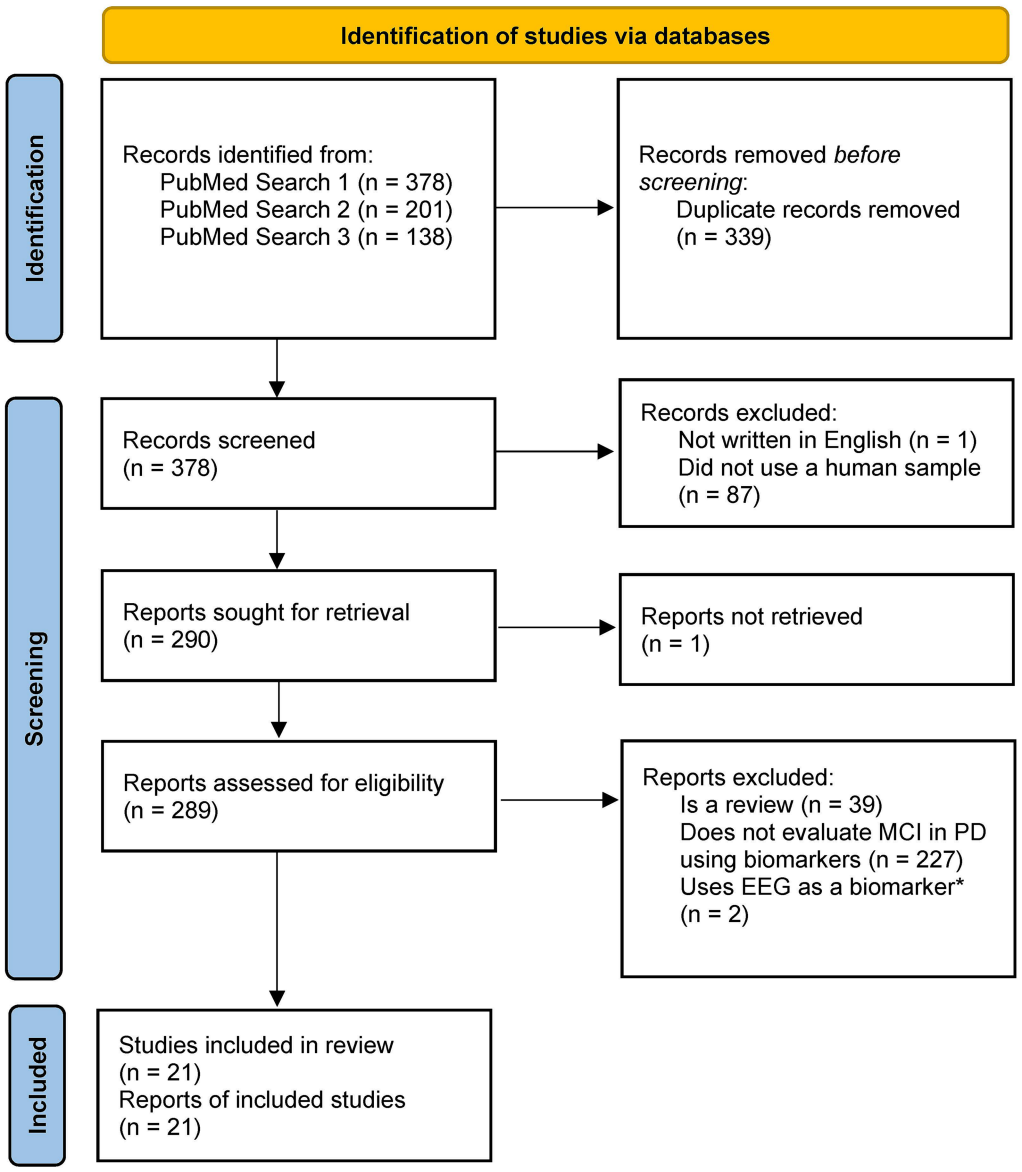
PRISMA diagram for study selection. *We decided to exclude studies using EEG as the main focus investigating the neuroimaging biomarkers was the application of MRI and PET. Template from: Page et al. (2021).

We created multiple searches through the PubMed database^1^ to determine the existing research on the use of biomarkers and machine learning to detect changes in cognition in subjects with PD. Our first search, which resulted in *N* = 289 English language articles pertaining to humans, aimed to provide context and background for the study. Only a few articles from this search focused on cognitive impairment and PD, which prompted subsequent searches. The second search (*N* = 160 English language articles pertaining to humans) was limited to require mention of PD or movement disorders, and the third search (*N* = 104 English language articles pertaining to humans) was further limited to articles discussing PD. The search keywords for the three searches are described in Table 1. Articles from the second and third searches were critically analyzed, and relevant articles were verified for scientific integrity by evaluating their place in subsequent literature and determining corroboration and support for claims. The inclusion criteria for eligible studies were as follows: (1) published in English; (2) is a research article, not a review article; (3) used a sample of human subjects, including subjects who were clinically diagnosed with PD; and (4) investigated changes in at least one biomarker (i.e., neuroimaging, biofluids, or clinical symptoms) to detect cognitive impairment in subjects with PD. The articles that passed these inclusion criteria were critically investigated and discussed in this study (*N* = 21) (Figure 1).

**Table 1:** Keywords for PubMed searches.

Search #	Keywords
1	(Parkinson’s disease OR movement disorders OR neurodegeneration) AND (neuroimaging OR biomarkers OR biofluids OR blood or cerebrospinal fluid) AND (machine learning OR deep learning OR artificial intelligence) AND (mild cognitive impairment OR dementia OR cognitive impairment)
2	(Parkinson’s disease OR movement disorders) AND (neuroimaging OR biomarkers OR biofluids OR blood or cerebrospinal fluid) AND (machine learning OR deep learning OR artificial intelligence) AND (mild cognitive impairment OR dementia OR cognitive impairment)
3	(Parkinson’s disease) AND (neuroimaging OR biomarkers OR biofluids OR blood or cerebrospinal fluid) AND (machine learning OR deep learning OR artificial intelligence) AND (mild cognitive impairment OR dementia OR cognitive impairment)

## 3 Machine learning techniques

### 3.1 Metrics

To evaluate the performance of machine learning techniques and interpret output, several accuracy metrics are used, including sensitivity, specificity, and area under the receiver operating curve (AUC). Sensitivity is defined as the “ability of a test to correctly classify an individual as diseased,” and is calculated as (# of true positives)/(# of true positives + # of false negatives) (Parikh et al., 2008). In comparison, specificity is defined as “the ability of a test to correctly classify an individual as disease-free,” and this metric is calculated as (# of true negatives)/(# of true negatives + # of false positives) (Parikh et al., 2008). A sensitivity of 100% would indicate that the test can detect all positive cases, while a specificity of 100% would indicate that the test can correctly classify all disease-free cases. These two metrics tend to be inversely proportional (as sensitivity increases, specificity decreases, and vice versa) (Parikh et al., 2008). The AUC uses randomized thresholds for the variable level required to classify subjects as positive to determine sensitivity and specificity. For example, a threshold of 0 used for a variable ranging from 0 to 1, where subjects above the threshold are classified as positive, would result in sensitivity of 100% and specificity of 0%. Inversely, a threshold of 1 would result in sensitivity of 0% and specificity of 100%. After determining the sensitivity and specificity for the thresholds, the area under the curve created by plotting sensitivity and (1-specificity) can be used to measure the model performance. As multiple iterations of thresholds are used for the AUC, it can be more accurate than sensitivity and specificity (Dennis and Strafella, 2024). The minimum sensitivity and specificity additionally depend on the disease prevalence. With low prevalence (e.g., 1%), the minimum sensitivity and specificity to achieve moderate diagnostic performance (for instance, 60% of patients with a positive result are positive) is 99–100% (Loh et al., 2021). With high prevalence (e.g., 60%), to achieve high diagnostic performance (e.g., 80–95% of patients with a positive result are positive), the minimum sensitivity ranges from 80 to 99% and the minimum specificity ranges from 70 to 95% (Loh et al., 2021). As most research applies data with 40–60% disease prevalence, a threshold of 80% for sensitivity and specificity would be appropriate to determine whether the study achieved high diagnostic performance (Loh et al., 2021). Based on this, a threshold of 80% for the AUC would also be appropriate.

### 3.2 Models

Training a machine learning-based model and testing the model on the same data can lead to inaccurately high model performance, and the model may perform well for that testing set but not be able to be generalized to other datasets. This is called overfitting; to avoid this issue, a training/testing split can be used, where a percentage of the data is used for training and the remainder is used for testing. Most researchers use 70–80% for training and 20–30% for testing. A technique that is commonly applied to avoid overfitting and to create a training/testing split is cross-validation, specifically *k*-fold cross-validation, where *k* is an integer (typically 5 or 10) selected by the researchers. In this technique, the dataset is split into *k* groups, and for *k* iterations, models are trained on *k*-1 groups of data and tested on the remaining held-out group. Higher levels of *k* correspond to more groups. Leave-one-out cross-validation (LOOCV) is a version of *k*-fold cross-validation where *k* is the size of the dataset (Dennis and Strafella, 2024). For instance, if there are 100 subjects in a dataset, the models would be trained 100 times with a different subject’s data as test data per iteration. Both techniques avoid training and testing on the same data, which can cause erroneous performance.

Another issue to consider while classifying using machine learning techniques is the number of variables (also called features or dimensions; dimensionality increases as the number of features increases) included in the dataset. Many techniques are prone to overfitting when numerous features are applied for classification, and not all features are important for the model, so techniques for feature extraction and selection are necessary. Principal component analysis (PCA) is one such technique; it uses multiple iterations to determine the pairs of variables that cause the most variance. Pairs with eigenvalues (a representation of total amount of variance that can be explained by the variables) >1 are included while the rest are excluded. Similar techniques include recursive feature elimination (RFE), which sorts variables by utility and removes the variables with low rankings, and RRELIEFF, which uses feature weights to detect features that are statistically relevant and pass the relevancy threshold (Dennis and Strafella, 2024; Ricciardi et al., 2020; Guyon et al., 2002; Kira and Rendell, 1992; Ramezani et al., 2021). Elastic Net-Based Feature Consensus Ranking (ENFCR) is a feature ranking algorithm that employs randomized train/test splitting and multiple ElasticNets (a type of generalized linear model discussed later) to select features, and then ranks features by selection frequency (Huang et al., 2024; Yu et al., 2020). Linear discriminant analysis (LDA) is a statistical-based pattern identification method to identify a vector of coefficients for the linear classification function, with the aim of maximizing the distance between classes and minimizing the distance within classes for the features (Ricciardi et al., 2020). This can be applied for both feature selection and classification.

Some simple machine learning techniques frequently used for classification include linear regression and logistic regression. Linear regression uses the input data to plot a linear relationship between variables; thus, it is most effective in continuous data with a linear correlation. Logistic regression is similar, except it uses a sigmoidal curve instead of a linear function (Dennis and Strafella, 2024; Choi et al., 2020). Generalized linear models (GLM), such as ElasticNet, are an expansion of linear regression, where the function of the outcome, or link function, can vary on covariates other than the predictor variables (Arnold et al., 2021; Harvey et al., 2022). This allows for more accurate model performance.

The neural network is based on a simple machine learning algorithm termed a perceptron. This method uses a structure similar to logistic regression, except it provides the class associations (e.g., PD) and not the probability of a classification (e.g., 70% probability of PD). When multiple perceptions are combined, the resulting model is referred to as a neural network (NN) or artificial neural network (ANN). Most NNs use a feedforward nature, where information flows from the input layer to the output layer. Additionally, they typically contain one layer of input nodes and one layer of output nodes, with simple NNs containing 0–3 hidden layers for classification, and more complicated NNs (termed deep neural networks) can have hundreds of layers. Convolutional neural networks are a type of NN created specifically for image processing, as they use patches of an image as input instead of single pixels, which preserves the spatial context. This allows for more efficient and accurate classification, since the image itself can be used as input (Dennis and Strafella, 2024; Choi et al., 2020).

One technique commonly used in classification that has been adapted to improve model performance and accuracy is the decision tree (DT). This method works by using the value of an input variable to divide the original set of data into subsets, which can then be further divided until the subset consists entirely of one class of subjects (i.e., PD). J48 is the Java implementation of the C4.5 DT proposed by Quinlan (Quinlan, 2014), which creates DTs using information entropy (entropy measures the uncertainty associated with a variable). The use of information entropy and pruning improves on the original DT technique. Because the DT technique can easily lead to overfitting by applying too many variables, the random forest (RF) technique has been developed as an extension. In this adaptation, multiple DTs are created based on different input variables (chosen from a random selection of variables), and majority vote is used for the final classification (Choi et al., 2020). When the input variable is chosen from all variables, this is a variety of RF named bagged trees (Dianati-Nasab et al., 2023). An alternative to RF is Cforest, which applies a permutation test for significance of variables instead of focusing on maximizing variance accounted for or information (Harvey et al., 2022). AdaBoost (ADA-B) is an ensemble learning technique similar to RF that determines weights for variables and adjusts them across multiple iterations of DTs for the best performance (Dietterich, 2000). Another type of DT frequently used is the gradient-boosting tree. Gradient boosting is an ensemble method of classification based on DTs that adds additional predictors by stages. Multiple derivative techniques have been created based on this technique, including XGBoost (extreme gradient boosting) and LightGBM (light gradient boosting machine) (Gao et al., 2018).

Two instance-based classification algorithms, which compare new data instances with data from the training set, that are frequently used include *k*-nearest neighbors (KNN) and support vector machine (SVM). KNN uses a pre-defined number *k* to define groups of *k* similar samples (where similarity is measured by the distance between groups) (Amboni et al., 2022). In contrast, SVM works by determining the hyperplane (a line in a high-dimensional space) able to separate the data into its classes (for instance, PD or HC). If only two variables are present, a simple straight line is sufficient, however, as dimensionality increases, this divider must consider these additional variables. Because of this, using SVM with a high dimensionality dataset can be problematic. The hyperplane is selected so that the maximum distance from the data of one class and the hyperplane is used (Dennis and Strafella, 2024; Noble, 2006). Multiple kernels are available to be used as basis for the hyperplane, including linear kernels and non-linear kernels (gaussian, quadratic, etc.) (Savas and Dovis, 2019). Based on the mechanism of the SVM, it can only handle binary classification; to classify between *n* classes, (*n*-1)! Different SVMs must be trained (Dennis and Strafella, 2024; Noble, 2006). SVM Regression (SVR) is an adaptation of this technique that is applied in regression, instead of classification. Because of the possibility of overfitting while using SVM, combining this model with feature selection may improve the classification. From this analysis, using a SVM, RF, or NN structure for classification combined with a feature selection technique would be most appropriate for detecting MCI in PD.

## 4 Biomarkers able to predict MCI and dementia in PD

### 4.1 Neuroimaging

Numerous studies have explored the application of machine learning techniques to detect progression of cognitive impairment in PD. A majority of researchers have focused on using neuroimaging as a biomarker. Neuroimaging modalities can be classified as structural, where the modality reflects structural changes such as atrophy, or functional, where the modality aims to show changes in the function of the brain. As both of these imaging types have value in diagnosis and prediction, we will review literature on detecting MCI in PD using (Marras et al., 2018) structural and (Song et al., 2022) functional neuroimaging with magnetic resonance imaging (MRI) and molecular imaging.

#### 4.1.1 Structural imaging

Diffusion tensor imaging (DTI) is one frequently used type of structural MRI for white matter. In Yu et al. (2023), the researchers aimed to apply data from DTI to distinguish HC (*n* = 30), PD with MCI (*n* = 30) and PD subjects with normal cognition (*n* = 40) from each other. Data was split into a training set (70%) and validation set (30%), and 10-fold cross-validation was applied. A feature importance analysis was completed using Shapley additive explanations (SHAP), a method for interpreting the output of the machine learning algorithms (Ning et al., 2022). SHAP found that the most important feature was the fractional anisotropy (FA) value of the anterior portion of the right inferior fronto-occipital fasciculus (IFOF). The SVM technique was used for classification. When the model was applied to differentiate PD with normal cognition (NC) from PD with MCI, accuracy, AUC, sensitivity, and specificity reached 79%, 0.80, 78, and 75%, respectively. The researchers found that the PD-MCI group exhibited significantly lower values of FA in segments of the left thalamic radiation and bilateral IFOF compared to PD-NC. Additionally, values of MD were significantly increased in segments of the bilateral corticospinal tract, corpus callosum major, and bilateral superior longitudinal fasciculus in PD-MCI. Additional research is required to determine when these biomarkers appear in the disease course and whether they should be used for MCI or more severe cognitive impairment.

Huang et al. (2024) used longitudinal data to observe differences in DTI data between PD subjects with normal cognition who converted to MCI (*n* = 33) and PD subjects who did not convert (*n* = 57). A total of 32 clinical and 90,774 neuroimaging-based features were extracted from the data. Due to the large number of features, feature selection was performed using *t*-tests (to remove features without significant between-group differences (*p* = 0.01)) and the ENFCR algorithm. This resulted in nine neuroimaging features and two clinical features. Additionally, subjects were split into a training and test set for the feature selection (*n* = 50 for training, *n* = 40 for testing) and to evaluate the model performance (*n* = 46 for training, *n* = 44 for testing). Multiple machine learning techniques were investigated, including SVM, KNN, Naïve Bayes (NB), and LDA. NB uses all the predictor variables in classification and assumes that all variables are independent of each other and the class. The reported accuracy metrics when clinical data is combined with neuroimaging were highest when using LDA (AUC: 0.85; Accuracy: 0.85; Sensitivity: 0.86; Specificity: 0.84) or NB (AUC: 0.84; Accuracy: 0.85; Sensitivity: 0.83; Specificity: 0.85). Similarly, when neuroimaging data was used alone, accuracy metrics were highest using LDA (AUC: 0.84, Accuracy: 0.82; Sensitivity: 0.85; Specificity: 0.82) or SVM (AUC: 0.83, Accuracy: 0.86, Sensitivity: 0.77, Specificity: 0.88). The use of LDA with combined neuroimaging and clinical biomarkers led to AUC, sensitivity, and specificity metrics all above 0.80/80%, while when biomarkers were used alone, these metrics were lower and, in some cases, below the threshold.

Chen et al. (2023) investigated the ability of features extracted from DTI to differentiate subjects with PD and MCI (*n* = 68) from subjects with PD and normal cognition (*n* = 52). From the imaging, 420 features were extracted [280 intravoxel (within voxel) and 140 intervoxel (between voxels)]. Because of the large feature size, RF feature selection, Spearman’s rank correlation analysis, and ShapleyVIC, which uses SHAP for variable importance (Savas and Dovis, 2019), were applied to determine the most important features. This analysis resulted in seven total features (two intravoxel, five intervoxel). Models were create based on the intravoxel, intervoxel, and combined features. The machine learning techniques applied were RF, DT, and XGBoost. In the intravoxel models, RF and XGBoost performed similarly with an accuracy of 75.00%. DT showed an accuracy of 54.17%. When the intervoxel features were used alone, accuracy decreased in comparison to the intravoxel features, with an accuracy of 66.67% in RF, 62.50% in XGBoost. For DT, the accuracy stayed constant, but the sensitivity decreased from 64.29 to 57.14%, and the specificity increased from 40.00 to 50.00%. In the combined models, RF had an accuracy of 75.00%, XGBoost resulted in an accuracy of 92.86%, and DT showed an accuracy of 66.67%. Using the combined features resulted in the highest performance across the model combinations. When XGBoost was applied with the intravoxel and intervoxel metrics combined, the accuracy was far above 80%. This is a noticeable difference compared to when intravoxel or intervoxel metrics were used individually (75.00 or 62.50% respectively). The findings from Chen et al. (2023) and Huang et al. (2024) suggest that combining biomarkers with different purposes may improve the ability of the model to diagnose MCI.

Using different structural MRI methodologies Morales et al. (2013) investigated the application of T1-weighted structural MRI to differentiate PD-NC (*n* = 16), PD-MCI (*n* = 15), PDD (*n* = 14) from each other. Stratified *k*-fold cross-validation was performed with *k* = 5, and four machine learning classification algorithms were compared: NB, filter selection Naïve Bayes (FSNB), correlation-based with feature subset selection - Naïve Bayes (CFS-NB), and SVM. FSNB is an adaption of NB that tests the independence between the variable and the class before using NB for classification. CFS-NB similarly ranks features by their correlation to the class and then performs NB classification.

When PDD subjects were differentiated from PD-NC subjects, the highest performing model was achieved by CFS-NB with an accuracy of 97.00 ± 6.74%, sensitivity of 93.33 ± 14.91%, and specificity of 100.00 ± 0.00%. When NB was used to differentiate PDD from PD-MCI, an accuracy of 96.55 ± 7.85%, sensitivity of 92.33 ± 14.91%, and specificity of 100.00 ± 0.00% were reported, while when FSNB was applied, an accuracy of 96.66 ± 10.33%, sensitivity of 92.00 ± 14.91%, and specificity of 100.00 ± 0.00% were reported. When PD-MCI was differentiated from PD-NC, the highest performing technique was CFS-NB with accuracy of 90.09 ± 8.40%, sensitivity of 93.00 ± 14.91%, and specificity of 88.33 ± 16.24%. When all three subject groups were differentiated using FSNB, accuracy reached 70.00 ± 26.66%, sensitivity reached 70.00 ± 26.66%, and specificity reached 85.56 ± 8.42%. Based on this, performance is better when classification is performed separately for each comparison (PDD/PD-NC, PDD/PD-MCI, or PD-MCI/PD-NC) as compared to when models attempt to distinguish all three classes (PDD/PD-MCI/PD-NC). Models performed best when PDD subjects were differentiated from other subjects, which is justifiable, as these subjects had the most severe level of cognitive impairment and would have noticeable differences in neuroimaging (Morales et al., 2013).

Nguyen et al. (2020) conducted a longitudinal assessment of structural T1-weighted MRI to determine which regions of the brain correlated to MoCA scores in subjects with PD (*n* = 74) and HC (*n* = 42) and use these features to classify subjects as NC or MCI. Feature selection was used through Spearman’s rank correlation (features with *p*-values over 0.05 were removed), and two classifiers were compared: a logistic regression model, and a deep learning-based autoencoder model. Autoencoders are algorithms that compress the input data and use a reduced representation of the input data to minimize loss of information; the researchers proposed that a model based on this framework would perform with higher accuracy when compared to logistic regression (Nguyen et al., 2020). Five-fold cross-validation was used to split the data. The features that contributed most to the model included the pallidum and right substantia nigra. The highest performances achieved by the models were using neuroimaging metrics from year 4; the autoencoder performed with 85% accuracy, 100% sensitivity, and 80% specificity, while the logistic regression model performed with ~75% accuracy, ~55% sensitivity, and ~ 85% specificity. The considerable difference between the autoencoder and logistic regression model suggests that complexity of a machine learning technique may correlate with performance of a model.

Quantitative susceptibility mapping (QSM) (MRI-derived) was used by Shibata et al. (2022) to classify subjects with PD as having MCI (Internal Set: *n* = 61; External Set: *n* = 22) or NC (Internal Set: *n* = 59; External Set: *n* = 21). For the machine learning techniques, RF, extreme gradient boosting, and light gradient boosting were used. Additionally, 10-fold cross-validation was applied. In both the internal and external sets, the RF model showed the highest performance (Internal: accuracy and AUC of 80.0 ± 16.3% and 0.84 ± 0.17; External: accuracy and AUC of 79.1% and 0.78). These results suggest that QSM can be applied to support diagnosis of MCI in PD.

Ramezani et al. (2021) explored the relationship between a combination of neuroimaging (structural T1-weighted MRI), clinical, and genetic features (102 features) and global cognition in subjects with PD (*n* = 101). RRELIEFF feature selection was applied to the features, which led to the top 11 features being used for classification, and nested LOOCV was used for the training/testing split. Support vector regression (SVR) was used as the classifier. The neuroimaging features used were five measures of cortical thickness (left entorhinal cortex, right parahippocampal cortex, right rostral anterior cingulate cortex, left middle temporal cortex, and right transverse temporal cortex) and right caudate volume. Other features included sex, MDS Unified Parkinson’s Disease Rating Scale (MDS-UPDRS) Part III, years of education, Edinburgh Handedness Inventory (EHI), and rs894280 (gene). These features resulted in a correlation coefficient of 0.54 and mean absolute error of 0.39. This indicates that there is a slight proportional relationship between the features and cognition, and the predictions tend to be ~0.39 points away from the cognitive score.

McFall et al. (2023) explored predicting onset of PDD in a group of subjects with PD (*n* = 48) using multi-modal predictors, including neuroimaging (structural MRI), blood-based biomarkers, and clinical characteristics. The total number of features from all modalities was 38. Stratified 3-fold cross-validation was applied. To determine which machine learning technique to apply, logistic regression, RF, and gradient boosting algorithms were applied. Additionally, Tree SHAP was applied as a method for interpreting the output of the machine learning algorithms. RF had the highest performance when used for classification, resulting in an AUC of 0.85 (95% CI 0.83–0.86) and accuracy of 81% (95% CI 80–82%). The variables were sorted in order of most to least important, and the top 10 predictors explain 62.5% of the model’s decision making. This includes gait, Trail A and B, and the volume of the third ventricle. The gradient boosting model additionally noted creatinine as an important biomarker. Since the top predictors include both clinical, imaging-based, and biofluid-based biomarkers, a combination for these biomarkers may be necessary for efficient diagnosis. All studies discussed in this section are discussed in Table 2.

**Table 2:** Main findings from articles using structural imaging.

Paper	Population: Sample size	Machine learning technique; cross-validation, training-test split; feature selection (number of features)	Biomarkers	Metrics collected	Metric values	Other findings
Yu et al. (2023)	HC: 30 PD-MCI: 30 PD-NC: 40	SHAP, SVM; 10-fold cross-validation, training: 70% Validation: 30%; No–feature importance (not reported)	DTI (FA; MD)	Accuracy, AUC, sensitivity, specificity	79%, 0.80, 78, 75%	The most important feature was the FA value of the anterior portion of the right IFOF.
Huang et al. (2024)	PD-NC: 57 PD-MCI: 33	SVM, KNN, NB, LDA; Feature selection: training: 50 testing: 40 Model evaluation: Training: 46 Testing: 44; yes (90,806 features to 11 features)	DTI (structural connectivity); MDS-UPDRS III, age	Accuracy, AUC, Sensitivity, Specificity	Combined Model, LDA: 85%, 0.85, 86, 84% Combined Model, NB: 85%, 0.84, 83, 85% Neuroimaging—LDA: 82%, 0.84, 85, 82% Neuroimaging—SVM: 86%, 0.83, 77, 88%	
Chen et al. (2023)	PD-NC: 52 PD-MCI: 68	RF, DT, XGBoost; None; yes (420 features to 7 features)	DTI (intravoxel, intervoxel)	Accuracy	RF - 75.00% XGBoost - 92.86% DT - 66.67%	
Morales et al. (2013)	PD-NC: 16 PD-MCI: 15 PDD: 14	NB, FSNB, CFS-NB, SVM; 5-fold cross-validation; yes (not reported)	T1-weighted structural MRI	Accuracy	PD-NC/PD-D, CFS-NB: 97.00 ± 6.74% PD-MCI/PD-D, NB: 96.55 ± 7.85% PD-NC/PD-MCI, CSF-NB: 90.09 ± 8.40% PD-NC/PD-MCI/PD-D, FSNB: 70.00 ± 26.66%	
Nguyen et al. (2020)	PD: 116 HC: 42	DL autoencoder, LR; 5-fold cross-validation; Yes (not reported)	T1-weighted structural MRI	Accuracy, Sensitivity, Specificity	DL: 85100, 80% LR: ~75%, ~55%, ~85%	
Shibata et al. (2022)	Internal: PD-NC: 59 PD-MCI: 61 External: PD-NC: 21 PD-MCI: 22	RF, extreme and light gradient boosting; 10-fold cross-validation; no (not reported)	QSM (MRI-derived)	Accuracy, AUC	Internal Set: 80.0 ± 16.3%, 0.84 ± 0.17 External Set: 79.1%, 0.78	
Ramezani et al. (2021)	PD: 101	SVR, RRELIEFF; Nested LOOCV; Yes (102 features to 11 features)	Features from structural T1-weighted MRI; clinical features; cognition; genetic features	Correlation coefficient, mean absolute error	0.54, 0.39	
McFall et al. (2023)	PD: 48	RF, LR, gradient boosting; 3-fold cross-validation; no (38 features)	Features from structural T1-weighted MRI; clinical features; blood-based features;	Accuracy, AUC	RF: 81% (95% CI 80–82%), 0.85 (95% CI 0.83–0.86)	
Abós et al. (2017)	HC: 38 PD-NC: 60 PD-MCI: 35	SVM, LR; LOOCV; yes (89 features to 21 features)	Features from T1-weighted structural MRI and RS-fMRI	Accuracy, AUC	SVM, LOOCV: 82.6% ± 3.9%, 0.88 ± 0.01	
Almgren et al. (2023)	PD: 213	SVR; LOOCV; yes (90 features to 12 features)	Features from T1-weighted structural MRI; clinical features; CSF-based features	N/A	N/A	The best model reported positive correlations between MoCA scores and beta-amyloid (*p* = 0.018) and negative correlations between MoCA scores and baseline cognition (*p* = 0.00004) and total tau (*p* = 0.049).

HC, healthy controls; PD-MCI, Parkinson’s disease with mild cognitive impairment; PD-NC, Parkinson’s disease with normal cognition; PD, Parkinson’s disease; PDD, Parkinson’s disease with dementia; SVM, support vector machine; KNN, k-nearest neighbors; NB, Naïve Bayes; LDA, linear discriminant analysis; RF, random forest; DT, decision tree; FSNB - filter selection Naïve Bayes; CFS-NB - correlation-based with feature subset selection, Naïve Bayes; DL, deep learning; LR, logistic regression; SVR, support vector regression; LOOCV, leave-one-out cross-validation; DTI, diffusion tensor imaging; QSM, quantitative susceptibility mapping; CSF, cerebrospinal fluid; FA, fractional anisotropy; IFOF, inferior fronto-occipital fasciculus; AUC, area under the receiver operating curve.

#### 4.1.2 Functional imaging

Fiorenzato et al. (2024) used functional MRI (fMRI) to distinguish PD-NC (*n* = 52), PD-MCI (*n* = 46), PDD (*n* = 20) and HC (*n* = 35) subjects. Feature selection was performed on the fMRI data (originally 595 features), which lead to 30 selected features for fractional amplitude of low-frequency fluctuations (fALFF) and 49 selected features for fractal dimension (FD). Multiple machine learning techniques were investigated, using five-fold cross-validation, including NN, SVM, RF, GradientBoost, and KNN. According to the researchers, when using fALFF features, models trained by a vote from NN, SVM, and RF classifiers demonstrated the highest accuracy (62.2 ± 1.4%) By comparison, when using FD as features, models trained using SVM demonstrated the highest accuracy when compared to other techniques (76 ± 1.6%), and this accuracy value was also much higher than when using fALFF.

Abós et al. (2017) aimed to apply T1-weighted structural MRI and resting (rs)-fMRI to detect MCI in PD. The training sample included HC (*n* = 38), PD-NC (*n* = 43), and PD-MCI (*n* = 27), while the validation sample included PD-NC (*n* = 17) and PD-MCI (*n* = 8). Feature selection was applied to avoid overfitting through the randomized logistic regression algorithm, which combines logistic regression with randomization of data, and the LOOCV technique (McFall et al., 2023). This technique selected 89 edges as features, and 21 of these features were selected in at least 80% of the iterations of LOOCV. Results from using LOOCV on the training data with SVM as the classification algorithm were compared with results using the validation sample and SVM, with only the 21 selected features. A mean accuracy of 82.6 ± 3.9% and mean AUC of 0.88 ± 0.01 was achieved using the first approach (when the PD-NC and PD-MCI subject groups were isolated, the mean accuracies were 82.6 ± 3.5% and 82.6 ± 4.3%, respectively). In contrast, the second approach resulted in an accuracy of 80% (PD-NC: 76.5%, PD-MCI: 87.5%) and an AUC of 0.81. Based on this, the first approach should be used in the PD-NC group, as this had a higher accuracy, and similarly the second approach should be used in the PD-MCI group. This research suggests that fMRI can be used to detect MCI and dementia in PD. Additionally, since models created by Abós et al. (2017) performed better than models created by Fiorenzato et al. (2024), and these models resulted in accuracy metrics above 80%, the use of multiple imaging modalities is further supported.

In Cengiz et al. (2022) and Marras et al. (2018) H-magnetic resonance spectroscopic imaging (1H-MRSI) was collected with the aim of differentiating PD subjects with MCI (*n* = 34) from NC (*n* = 26), and from HC (*n* = 16). Multiple machine learning techniques were tested for model performance, including KNN, bagged trees, and SVM with a fine Gaussian kernel. Results were reported for the highest-performing technique, and five-fold cross-validation was used to avoid overfitting. Metabolic ratios were used as features in the machine learning-based models. When fine Gaussian SVM was used to differentiate PD-NC and PD-MCI, as it had the highest performance out of the classifiers, accuracy, sensitivity, and specificity were reported as 77.3, 63.6, and 69.7%. Similarly, when bagged trees was used for the HC/PD-NC and HC/PD-MCI comparisons, accuracy, sensitivity, and specificity were 86.5, 73.1, and 84.6%, respectively, for HC/PD-NC, and accuracy, sensitivity, and specificity were 86.4, 72.7, and 81.8%, respectively, for HC/PD-MCI. The higher performance when differentiating HC from PD-NC or PD-MCI as compared to differentiating PD-NC from PD-MCI may be supported by differences in biomarkers caused by presence of PD. Additionally, the model performance decreased when HC was differentiated from PD-MCI, which suggests that 1H-MRSI imaging of subjects with PD-MCI may be more similar to HC than the 1H-MRSI imaging of subjects with PD without MCI.

A recently developed hemodynamic-based technique for functional imaging is functional near-infrared spectroscopy (fNIRS). Shu et al. (2024) investigated the use of fNIRS to distinguish PD subjects with MCI (*n* = 20) from HC (*n* = 34). Graph frequency analysis (GFA) was applied to deconstruct the imaging and extract features. Specifically, GFA analyzes the graph in the graph frequency domain and the graph spectrum of the functional brain networks. The two main features extracted were total variation (TV) (*n* = 18), which measures differences of signals for each edge in a specific region, and weighted zero crossings (WZC) (*n* = 6), which quantifies the spatial variability of the graph eigenvectors globally. SVM and LOOCV were applied for classification. Using TV alone resulted in accuracy and F1 score of 62.7% and 0.773, while using WZC alone resulted in accuracy of 79.6% and F1 score of 0.849. When all features were combined, accuracy and F1 score of 83.3% and 0.877 were achieved, which is a significant increase from TV or WZC alone and continues to support the use of multiple features in creating classification models. Based on this, fNIRS may have utility in diagnosing PD and/or MCI.

Various studies have applied positron emission tomography (PET) for diagnosing PD, so Choi et al. (2020) aimed to develop a model that could be used for identifying conversion from PD to PDD. This model was trained on a dataset of subjects with Alzheimer’s disease (AD) (*n* = 243) and HC (*n* = 393) and tested on a dataset of PD subjects [with dementia (*n* = 13); NC (*n* = 49)]. The features (*n* = 128 per subject) processed by the model were extracted from FDG PET, and a CNN framework was used as the basis for the model. This model resulted in an AUC of 0.81 (95% CI 0.68–0.94) when the model was tested on data from PD subjects.

As research supports that levels of beta-amyloid-42 (Aβ42) in cerebrospinal fluid (CSF) can be used to predict cognitive decline, Amboni et al. (2022) investigated whether Aβ42 PET imaging could be used to differentiate subjects with PD and MCI (*n* = 33) from subjects with PD and no MCI (*n* = 42). Two sets of features were used to create machine learning-based models. The first model used clinical characteristics and features from gait analysis, and the second models used the top five features from the first model and features from PET. Model “2A” comprised of the averaged standardized uptake values (SUVs) of all nine regions from PET, while Model “2B” comprised of the averaged SUVs of the cortical regions alone. LOOCV was used for the second models, and 10-fold cross-validation was used in the first model. Multiple machine learning techniques were used to create separate models, specifically RF, J48, and ADA-B as tree-based algorithms, and SVM and KNN as instance-based algorithms. For the first model, the highest performing model techniques were SVM and RF, with accuracy, sensitivity, specificity, and AUC of 80.0, 72.7, 85.7%, 0.792 for SVM and 73.3, 66.7, 78.6%, 0.722 for RF. In Model “2A,” the highest performers were SVM (72.2, 73.7, 70.6%, 0.721) and J48 (72.2, 73.7, 70.6%, 0.621), and in Model “2B,” the highest performers were SVM (75.0, 73.7, 76.5%, 0.751) and J48 (69.4, 68.4, 70.6%, 0.636). In all models, SVM performed with the highest performance. However, performance deceased when Aβ42 PET features were used as compared to when clinical features were applied. When SVM was used as a classifier, Model “2B” performed with higher accuracy metrics than Model “2A,” and the model using clinical metrics performed with highest accuracy. This suggests that combining clinical metrics with the neuroimaging metrics may further improve the accuracy metrics. All studies discussed in this section are discussed in Table 3.

**Table 3:** Main findings from articles using functional imaging.

Paper	Population: sample size	Machine learning technique; cross-validation, training-test split; feature selection (number of features)	Biomarkers	Metrics collected	Metric values	Other findings
Fiorenzato et al. (2024)	HC: 35 PD-NC: 52 PD-MCI: 46 PDD: 20	NN, SVM, RF, GradientBoost, KNN; 5-fold cross-validation; yes (595 features to 79 features)	fMRI (FD, fALFF)	Accuracy	fALFF, NN, SVM, RF: 62.2 ± 1.4% FD, SVM: 76 ± 1.6%	
Abós et al. (2017)	HC: 38 PD-NC: 60 PD-MCI: 35	SVM, LR; LOOCV; Yes (89 features to 21 features)	Features from T1-weighted structural MRI and resting state-fMRI	Accuracy, AUC	SVM, LOOCV: 82.6% ± 3.9%, 0.88 ± 0.01	
Cengiz et al. (2022)	PD-MCI: 34 PD-NC: 20 HC: 16	KNN, bagged trees, SVM; 5-fold cross-validation; No (not reported)	3D 1H-MRSI	Accuracy, Sensitivity, Specificity	PD-NC/PD-MCI, SVM: 77.3, 63.6, 69.7% HC/PD-NC, bagged trees: 86.5, 73.1, 84.6% HC/PD-MCI, bagged trees: 86.4, 72.7, 81.8%	
Shu et al. (2024)	PD-MCI: 20 HC: 34	SVM; LOOCV; No (24 features)	fNIRS	Accuracy, F1 score	TV: 62.7%, 0.773 WZC: 79.6%, 0.849 Combined: 83.3%, 0.877	
Choi et al. (2020)	AD: 243 HC: 393 PDD: 13 PD-NC: 49	CNN; trained on AD/HC data, tested on PD-NC/PDD data; no (128 features)	FDG PET	AUC	PD subjects: 0.81 (95% CI 0.68–0.94)	
Amboni et al. (2022)	PD-NC: 42 PD-MCI: 33	RF, J48, ADA-B, SVM, KNN; LOOCV; No (25 features)	Beta-amyloid-42 PET; clinical features	Accuracy, AUC, sensitivity, specificity	Clinical features: 80.0%, 0.792, 72.7, 85.7% All PET features: 72.2%, 0.721, 73.7%, 70.6 Cortical PET features: 75.0%, 0.751, 73.7, 76.5%	All high performing models used SVM.

HC, healthy controls; PD-NC, Parkinson’s disease with normal cognition; PD-MCI, Parkinson’s disease with mild cognitive impairment; PD-D, Parkinson’s disease with dementia; AD, Alzheimer’s disease; NN: neural network; SVM, support vector machine; RF, random forest; KNN, k-nearest neighbors; LR, logistic regression; LOOCV, leave-one-out cross-validation; CNN, convolutional neural network; ADA-B, AdaBoost; FD, fractal dimension; fALFF, fractional amplitude of low-frequency fluctuations; AUC, area under the receiver operating curve.

### 4.2 Clinical

As symptoms can reflect the state of the disease, some studies have explored the use of clinical tests to predict onset of MCI. One such study created a model using clinical metrics, such as cognitive impairment, tremors, and neuropsychiatric measures to define and identify disease stages in PD (Severson et al., 2021). All subjects were diagnosed with PD (*n* = 433), and the sample was split into a training (*n* = 333) and testing (*n* = 82). Five-fold cross-validation was applied to the training data. No feature selection techniques were implemented; 82 features were used to determine the appropriate stages. As hidden Markov models (HMM), a type of computational model, can describe transitions between sequential data through forming stages, the researchers created a model using this structure to identify eight disease stages of PD. The final stage, stage 8, accounted for 56% of cases of MCI and 95% of dementia cases. This research suggests that artificial intelligence and machine learning techniques may be used to recognize and separate disease stages and severity through clinical measures, and that these technologies may be applied to the prediction of worsening cognitive impairment.

Brien et al. (2023) applied video-based eye tracking to differentiate HC (*n* = 106) from PD (PD-NC: *n* = 45, PD-MCI: *n* = 45, PDD: *n* = 20) and detect PD progression. Two sets of features were extracted for a total of 45 features: point estimates (21 features), which were estimates of mean values and rates, and functional estimates (24 features), which were functional summary features extracted from functional data analysis and functional PCA. Functional PCA is another version of PCA specifically for functional features. Nested 10-fold cross-validation was used to create the test set, and the machine-learning based model was based on three classifiers: SVM, RF, and logistic regression. The probability of PD for each classifier was collected and averaged to determine the final probability of PD and classify. The sensitivity, specificity, and AUC were 83, 78%, and 0.88, respectively, for the classification of HC/PD. The AUC was collected for each subset of PD subjects organized by cognitive ability: the HC/PD-NC, HC/PD-MCI, and HC/PDD classifications achieved AUCs of 0.84, 0.89, and 0.95. The increase in AUC as cognitive ability of the PD subjects decreased is justifiable, as the differences between HC and PD subjects would have arguably become more noticeable.

Harvey et al. (2022) completed a longitudinal assessment of cognitive function in subjects with PD, where clinical, biofluid, and genetic data were collected repeatedly. Subjects with PD were organized as NC (*n* = 67), MCI (*n* = 39), PDD (*n* = 43), or subjective cognitive decline (*n* = 60). Multiple machine learning techniques were assessed: RF, conditional inference forest (Cforest), SVM, and ElasticNet. A variable importance assessment was completed through RFE to determine which variables contribute to the high performing models, and 10-fold cross-validation was applied. When models were created to identify MCI converters, the combined model (28 features, using Cforest) achieved an accuracy, AUC, sensitivity, and specificity of 86.7%, 0.938, 71.9, and 96.1%, respectively. The accuracy, AUC, sensitivity, and specificity for the clinical model (11 features, using Cforest) were 85.5%, 0.930, 65.6, and 98.0%, respectively. In comparison, the accuracy, AUC, sensitivity, and specificity for the biofluid-based model (4 features, using ElasticNet) were 68.7%, 0.756, 62.5, and 72.5%, respectively. For models created to identify PDD converters, using the combined model (10 features, using SVM) resulted in an accuracy, AUC, sensitivity, and specificity of 81.9%, 0.862, 47.1, and 90.9%. In comparison, the clinical model (8 features, using RF) reached an accuracy, AUC, sensitivity, and specificity of 80.7%, 0.828, 47.1, and 89.4%. The biofluid model (5 features, ElasticNet) reached an accuracy, AUC, sensitivity, and specificity of 86.7%, 0.835, 47.1, and 97.0%. In models created for MCI, the highest performance was achieved by the combined model, although this model resulted in lower sensitivity, while in the models created for PDD, the highest performance was achieved by the biofluid model, with substandard sensitivity. The sensitivity was lower than the specificity in all models, which suggests that these biomarkers should be used to help rule out chances of conversion rather than diagnose. All studies discussed in this section are discussed in Table 4.

**Table 4:** Main findings from articles using clinical features.

Paper	Population: sample size	Machine learning technique; cross-validation, training-test split; feature selection (number of features)	Biomarkers	Metrics collected	Metric values	Other findings
Huang et al. (2024)	PD-NC: 57 PD-MCI: 33	SVM, KNN, NB, LDA; feature selection: Training: 50 Testing: 40 Model evaluation: Training: 46 Testing: 44; yes (90,806 features to 11 features)	DTI (structural connectivity); MDS-UPDRS III, age	Accuracy, AUC, sensitivity, specificity	Combined model, LDA: 85%, 0.85, 86, 84% Combined model, NB: 85%, 0.84, 83, 85% Neuroimaging—LDA: 82%, 0.84, 85, 82% Neuroimaging—SVM: 86%, 0.83, 77, 88%	
Ramezani et al. (2021)	PD: 101	SVR, RRELIEFF; Nested LOOCV; Yes (102 features to 11 features)	features from structural T1-weighted MRI; clinical features; cognition; genetic features	Correlation coefficient, mean absolute error	0.54, 0.39	
McFall et al. (2023)	PD: 48	RF, LR, gradient boosting; 3-fold cross-validation; no (38 features)	Features from structural T1-weighted MRI; clinical features; blood-based features;	Accuracy, AUC	RF: 81% (95% CI 80–82%), 0.85 (95% CI 0.83–0.86)	
Almgren et al. (2023)	PD: 213	SVR; LOOCV; Yes (90 features to 12 features)	Features from T1-weighted structural MRI; clinical features; CSF-based features	N/A	N/A	The best model reported positive correlations between MoCA scores and beta-amyloid (*p* = 0.018) and negative correlations between MoCA scores and baseline cognition (*p* = 0.00004) and total tau (*p* = 0.049).
Amboni et al. (2022)	PD-NC: 42 PD-MCI: 33	RF, J48, ADA-B, SVM, KNN; LOOCV; No (25 features)	Beta-amyloid-42 PET; Clinical features	Accuracy, AUC, Sensitivity, Specificity	Clinical features: 80.0%, 0.792, 72.7, 85.7% All PET features: 72.2%, 0.721, 73.7%, 70.6 Cortical PET features: 75.0%, 0.751, 73.7, 76.5%	All high performing models used SVM.
Severson et al. (2021)	PD: 316	HMM; 5-fold cross-validation; no (82 features)	Cognitive and motor functioning, Hoehn and Yahr score, death			The model was able to determine eight stages of PD, where stage 8 is the most severe for all but one symptoms (tremor); stage 5 is the most severe tremor.
Brien et al. (2023)	HC: 106 PD-NC: 45 PD-MCI: 45 PDD: 20	SVM, RF, LR; Nested 10-fold cross-validation; no (45 features)	Video-based eye tracking	AUC	HC/PD: 0.88, HC/PD-NC: 0.84 HC/PD-MCI: 0.89 HC/PDD: 0.95	
Harvey et al. (2022)	PD-NC: 67 PD-MCI: 39 PDD: 43	RF, Cforest, SVM, ElasticNet; 10-fold cross-validation; no (not reported)	Clinical features; Biofluid features; Genetic features; Cognition	Accuracy	MCI converters, Combined model, CForest: 86.7% MCI converters, clinical model, CForest: 85.5% MCI converters, Biofluid-Based Model, ElasticNet: 68.7% PDD converters, Combined model, SVM: 81.9% PDD converters, clinical model, RF: 80.7% PDD converters, Biofluid-Based Model, ElasticNet: 86.7%	
Chung et al. (2021)	HC: 46 PD: 116	ANN; 4-fold cross-validation; No (5 features)	Blood-based biomarkers (AB-42, tau, a-syn) Clinical features (sex, age)	Accuracy, AUC, sensitivity, specificity	91.3% 0.911, 100, 60.0%	The most effective biomarkers were tau and AB-42, with sex, age, and alpha-synuclein being less important.
Deng et al. (2023)	PD-MCI: 108 PD-NC: 98	ShapleyVIC; N/A No (41 features)	Blood-based features; clinical features; Genetic features	N/A	N/A	The clinical variables with the most importance to PD-MCI were years of education, hypertension, MDS-UPDRS Part III motor score and the blood-based variables were triglyceride and ApoA1.

PD-NC, Parkinson’s disease with normal cognition; PD-MCI, Parkinson’s disease with mild cognitive impairment; PD, Parkinson’s disease; HC, healthy controls; PD-D, Parkinson’s disease with dementia; SVM, support vector machine; KNN, k-nearest neighbors; NB, Naïve Bayes; LDA, linear discriminant analysis; SVR, support vector regression; LOOCV, leave-one-out cross-validation; RF, random forest; LR, logistic regression; ADA-B, AdaBoost; HMM, hidden Markov model; ANN, artificial neural network; CSF, cerebrospinal fluid; AB-42, beta-amyloid-42; AUC, area under the receiver operating curve.

### 4.3 Biofluids

Biofluids have frequently been used to distinguish subjects with PD from HC (Kelly et al., 2023), however when focusing on detecting MCI in PD, studies tend to focus on neuroimaging-based or clinical biomarkers instead of biofluid biomarkers. Despite this, some studies have explored the application of biomarkers common in AD to PD-MCI and PDD. For instance, prior research has suggested an association between cognitive impairment and levels of tau and Aβ42 in AD (Chung et al., 2021). Based on this, Chung et al. (2021) extracted extracellular vesicles from plasma to assess concentrations of Aβ42, tau, and alpha-synuclein and whether these concentrations could be applied to classify cognitive function in PD. The sample included 46 HC and 116 PD, and subjects with PD were grouped based on their cognitive function. The number of subjects in each cognitive group was not reported. An artificial neural network (ANN) was created based on the age, sex, tau, alpha-synuclein, and Aβ42, and four-fold cross-validation was used. The model performed with an accuracy of 91.3%, AUC of 0.911, precision of 90.0%, sensitivity of 100%, specificity of 60.0%. As there were much fewer HC subjects than PD subjects, the low specificity did not have a major effect on the accuracy. An analysis was also completed on the effect of each biomarker on the model’s predictions, and the most effective biomarkers were tau and Aβ42, with sex, age, and alpha-synuclein being less important.

Almgren et al. (2023) investigated the use of multimodal features, including CSF-based biomarkers, (such as total-tau, phosphorylated-tau, Aβ42, and alpha-synuclein) and MRI-based volumetric data to predict cognitive decline in subjects with PD (*n* = 213). Feature selection was applied to reduce the number of features from 90 features to 12 features. Ten-fold cross-validation was applied during the training process, and the most important biomarkers were determined by the number of folds that a biomarker appeared in. Based on this, the most important features were the MoCA, t-tau, p-tau, Aβ42, Geriatric Depression Scale, and State Trait Anxiety Inventory (STAI). Of these, statistically significant positive correlations were found between MoCA scores and Aβ42 (*p* = 0.018), while statistically significant negative correlations were found between MoCA scores and total tau (*p* = 0.049) and STAI (*p* = 0.042). The correlations between MoCA scores and Aβ42 and tau are justifiable, as these proteins are found at abnormal levels in Alzheimer’s disease and mild cognitive impairment. Additionally, the researchers found that CSF alpha-synuclein was an important feature, however, it was lower ranked as compared to other CSF biomarkers.

Lin et al. (2020) compared results from seven classifiers (NB, KNN, SVM, C4.5 DT, classification and regression trees (which uses the DT structure), RF, logistic regression) and LDA to differentiate PD-NC (*n* = 57), PD-MCI (*n* = 29), and PDD (*n* = 87) from each other based on Aβ42, beta-amyloid-40 (Aβ40), total-tau, phosphorylated-tau-181 (p-tau-181), and alpha-synuclein levels in plasma. LDA was able to reduce data dimensionality from 5 dimensions to 3 dimensions. LOOCV was additionally applied to split the data into a training and test set. Analysis revealed that levels of Aβ40 were lower in PD-NC compared to HC (*n* = 97), and levels of total-tau and p-tau-181 were significantly higher in PD when compared to HC. Additionally, alpha-synuclein levels was increased in all PD groups when compared to HC and were highest in subjects with PDD. The highest accuracy rate was approximately 68%, achieved by either NB or logistic regression. This lower accuracy rate compared to earlier articles combining biofluids with another biomarker (Harvey et al., 2022; McFall et al., 2023) suggests that using a combination of features improves the ability of the classification models to identify MCI in PD.

In addition, one article focused on ranking a series of blood-based, genetic, and clinical biomarkers by importance using ShapleyVIC-assisted variable selection to determine which variables are correlated to MCI in PD (Deng et al., 2023). The algorithm found 22 variables, out of 41 analyzed, that had significant importance when comparing subjects with PD and MCI (*n* = 108) and subjects with PD and normal cognition (*n* = 98). The clinical variables with the most importance to PD-MCI were years of education, hypertension, MDS-UPDRS Part III motor score and the blood-based variables were triglyceride and ApoA1. Prior research finds that higher levels of triglyceride and ApoA1 correlate to increased neuroinflammation, and that these levels may be associated with underlying Aβ42/tau pathology (Deng et al., 2023; Deng et al., 2022). The findings that Deng et al. (2023) reports suggest that levels of triglyceride and ApoA1 in blood may be therapeutic targets for MCI in PD.

These findings support the use of Aβ42 and tau for diagnostic and therapeutic targets, and that similar biomarkers linked to Aβ42/tau pathology should be investigated further through machine learning techniques (Chung et al., 2021; Almgren et al., 2023; Lin et al., 2020; Deng et al., 2023). All studies discussed in this section are discussed in Table 5.

**Table 5:** Main findings from articles using biofluid features.

Paper	Population: sample size	Machine learning technique; cross-validation, training-test split; feature selection (number of features)	Biomarkers	Metrics collected	Metric values	Other findings
McFall et al. (2023)	PD: 48	RF, LR, gradient boosting; 3-fold cross-validation; no (38 features)	Features from structural T1-weighted MRI; clinical features; blood-based features;	Accuracy, AUC	RF: 81% (95% CI 80–82%), 0.85 (95% CI 0.83–0.86)	
Almgren et al. (2023)	PD: 213	SVR; LOOCV; yes (90 features to 12 features)	Features from T1-weighted structural MRI; clinical features; CSF-based features	N/A	N/A	The best model reported positive correlations between MoCA scores and beta-amyloid (*p* = 0.018) and negative correlations between MoCA scores and baseline cognition (*p* = 0.00004) and total tau (*p* = 0.049).
Harvey et al. (2022)	PD-NC: 67 PD-MCI: 39 PDD: 43	RF, Cforest, SVM, ElasticNet; 10-fold cross-validation; No (not reported)	Clinical features; biofluid features; genetic features; cognition	Accuracy	MCI converters, Combined Model, CForest: 86.7% MCI converters, Clinical Model, CForest: 85.5% MCI converters, biofluid-based model, ElasticNet: 68.7% PDD converters, Combined Model, SVM: 81.9% PDD converters, clinical model, RF: 80.7% PDD converters, Biofluid-based model, ElasticNet: 86.7%	
Chung et al. (2021)	HC: 46 PD: 116	ANN; 4-fold cross-validation; No (five features)	Blood-based biomarkers (AB-42, tau, a-syn) clinical features (sex, age)	Accuracy, AUC, sensitivity, specificity	91.3% 0.911, 100, 60.0%	The most effective biomarkers were tau and AB-42, with sex, age, and alpha-synuclein being less important.
Lin et al. (2020)	PD-NC: 57 PD-MCI: 29 PDD: 87 HC: 97	NB, KNN, SVM, DT, CART, RF, LR, LDA; LOOCV; yes (5–3 features)	Blood-based biomarkers (AB-40, AB-42, t-tau, p-tau-181, a-syn)	Accuracy	NB or LR: ~68%	Levels of AB-40 were lower in PD-NC compared to HC. Levels of t-tau and p-tau-181 were significantly higher in PD compared to HC. Alpha-synuclein levels were higher in all PD groups when compared to HC and were highest in subjects with PDD
Deng et al. (2023)	PD-MCI: 108 PD-NC: 98	ShapleyVIC; N/A No (41 features)	Blood-based features; clinical features; genetic features	N/A	N/A	The clinical variables with the most importance to PD-MCI were years of education, hypertension, MDS-UPDRS Part III motor score and the blood-based variables were triglyceride and ApoA1.

PD, Parkinson’s disease; PD-NC, Parkinson’s disease with normal cognition; PD-MCI, Parkinson’s disease with mild cognitive impairment; PD-D, Parkinson’s disease with dementia; HC, healthy controls; SVR, support vector regression; LOOCV, leave-one-out cross-validation; RF, random forest; LR, logistic regression; SVM, support vector machine; ANN, artificial neural network; NB, Naïve Bayes; KNN, k-nearest neighbors; DT, decision tree; CART, classification and regression trees; LDA, linear discriminant analysis; CSF, cerebrospinal fluid; AB-42, beta-amyloid-42; AB-40, beta-amyloid-40; t-tau, total-tau; p-tau-181, phosphorylated-tau-181; a-syn, alpha-synuclein; AUC, area under the receiver operating curve.

## 5 Conclusion

Most prior research applying artificial intelligence to detection of MCI in subjects with PD focuses on neuroimaging biomarkers (*n* = 15), with structural (*n* = 10) and functional (*n* = 6) modalities both explored in articles. Similar amounts of research have been reported for clinical biomarkers (*n* = 8) and biofluid biomarkers (*n* = 6). In addition, *n* = 8 articles (35%) discussed combining biomarker types together, with neuroimaging, clinical, and biofluid biomarkers discussed in *n* = 5, *n* = 8, and *n* = 5 articles. Most studies were cross-sectional (*n* = 14), with *n* = 7 longitudinal studies. Since diagnosis may be supplemented by using multiple data points over a period of time to observe change, longitudinal studies may be more useful at determining a biomarker.

Out of all *n* = 21 articles, *n* = 17 articles reported metrics that can be compared to the 0.80/80% threshold, including sensitivity, specificity, and AUC. We noted whether these articles followed proper protocol for training and testing the models to determine if there was a correlation between an improper modeling protocol and metrics below the threshold. Based on this, *n* = 6 articles used both a training/testing split (through cross-validation or manual split) and feature selection. 4 (67%) of these articles (Huang et al., 2024; Morales et al., 2013; Nguyen et al., 2020; Abós et al., 2017) reported metrics above 80%, while *n* = 2 articles’ (Fiorenzato et al., 2024; Lin et al., 2020) results were below the threshold for successful classification. Of the remaining *n* = 11 studies, *n* = 10 did not apply feature selection and the other *n* = 1 did not apply the training/testing split. The former category contained *n* = 4 studies (40%) with metrics above 80% (Harvey et al., 2022; McFall et al., 2023; Shu et al., 2024; Brien et al., 2023) and *n* = 6 with substandard results (Amboni et al., 2022; Yu et al., 2023; Shibata et al., 2022; Cengiz et al., 2022; Choi et al., 2020; Chung et al., 2021). It is possible that the *n* = 1 study not using the training/testing split (Chen et al., 2023) reported one metric (accuracy) above the threshold due to erroneous reporting. Additionally, no articles used a training/validation/test split, which splits data into three sets instead of two (a training/test split), although *n* = 2 used an external set of data (Shibata et al., 2022; Choi et al., 2020) to validate the model performance and improve the model robustness. It is common when classification models are tested on the same data they are trained on, the results may be flawed, and the reporting may be inaccurate. Based on this analysis, metrics above the 0.80/80% threshold are correlated to a proper and thorough model creation protocol, and it is necessary to properly train and test models for a diagnostic algorithm to be identified.

The results from these studies suggest that applying biomarkers from different sources may improve the ability of the model to detect MCI. Specifically, Aβ42, tau, and alpha-synuclein may be used as biofluid biomarkers (Chung et al., 2021; Deng et al., 2023), and MRI (structural and functional) may be used as neuroimaging biomarkers (Nguyen et al., 2020; Abós et al., 2017). However, this analysis is limited, as there are few robust studies using artificial intelligence to identify MCI in subjects with PD. Because of this, the performance reported in these studies may be unreliable. Future directions should involve replication of these studies in different datasets to determine if these biomarkers are generalizable.

## Data Availability

The original contributions presented in the study are included in the article/supplementary material, further inquiries can be directed to the corresponding author.

## Footnotes

^1^ https://pubmed.ncbi.nlm.nih.gov/
